# Carotid Artery Stenting Using Five-French Distal Radial Vascular Access

**DOI:** 10.3390/diagnostics13071266

**Published:** 2023-03-27

**Authors:** Giuseppe Di Gioia, Luigi Salemme, Marco Ferrone, Angelo Cioppa, Grigore Popusoi, Armando Pucciarelli, Sebastiano Verdoliva, Michele Franzese, Simion Marga, Emanuele Barbato, Tullio Tesorio

**Affiliations:** 1 Catheterization Laboratory, Montevergine Clinic, 83013 Mercogliano, Italy; 2 Department of Radiology, Nicolae Testemitanu State University of Medicine and Pharmacy, MD-2000 Chișinău, Moldova; 3 Department of Clinical and Molecular Medicine, Sapienza University of Rome, 00189 Rome, Italy

**Keywords:** carotid artery disease, carotid stenting, radial artery

## Abstract

Carotid artery stenting (CAS) is usually performed through a femoral vascular access using 6–9 Fr guiding catheters. We investigated whether a systematic distal radial approach using 5 Fr guiding sheaths was a safe and effective alternative to transfemoral approach for CAS. From July 2020 to October 2022, two operators at our center systematically performed CAS using a 5 Fr distal radial approach in consecutive patients. The main endpoints of the study were procedural success via distal radial and via proximal or distal radial access. The learning curve was evaluated by comparing the first half of patients versus the second half of patients enrolled. Procedural data and 30-day clinical outcomes were collected. Fifty-one patients were prospectively enrolled. CAS was effectively performed via distal radial access in 45 patients (88%). Overall radial artery success was 92%. Distal radial CAS was successfully performed in 20 out of the first 25 patients enrolled (80%), and in 25 of the last 26 patients enrolled (96%; *p* = 0.07). Significantly less contrast was administered in the last 26 patients compared to the first 25 enrolled (110 (70, 140) mL vs. 120 (107, 150) mL; *p* = 0.045). Radial artery occlusion was reported in 1 patient (2%). Only 1 minor stroke (2%) was reported in-hospital and at 30-day follow-up. In conclusion, distal radial CAS using 5 Fr catheters was a safe procedure with a high success rate. The procedure had a relatively short learning curve in operators familiar with transfemoral CAS.

## 1. Introduction

Carotid artery stenting (CAS) is usually performed through a femoral vascular access using 6–9 Fr guiding catheters. Patients undergoing CAS are generally at higher risk for vascular-access-related complications and bleeding, since bigger-bore catheters tend to be used, as compared with coronary procedures, and carotid atherosclerosis is often associated with lower-limb arteriopathy [1].

Transradial CAS (TRCAS), on the other hand, is performed routinely in very few centers worldwide, and it is generally reserved for selective cases. In fact, thorough imaging of the aortic arch and supra-aortic vessels with computed tomography or magnetic resonance is considered to be mandatory for this approach. In particular, a Type 2 bovine arch with left internal carotid artery (LICA) stenosis represents an indication for TRCAS, whereas right ICA (RICA) stenosis with Type 2 or 3 aortic arch is considered to be a favorable anatomy for transradial access. Ultimately, lack of other viable peripheral vascular access represents an obligatory indication for TRCAS [2].

Radial access obtained at the level of the anatomic snuffbox (distal radial access) has been associated with significantly higher radial artery patency rates and shorter hemostasis time as compared with a proximal radial approach [3,4,5], but this has only been reported anecdotally for CAS [6]. Distal radial access for carotid interventions might be particularly convenient, since patients undergoing CAS often need multiple catheterizations due to atherosclerosis of other vascular districts. Moreover, short hemostasis time could potentially allow operators to perform these procedures without discontinuation of oral anticoagulation. Finally, radial access has been shown to reduce acute kidney injury (AKI) compared to femoral access due to a reduction in bleedings, vascular complications and renal cholesterol embolization [7,8]. The early mobilization of patients treated with CAS transradially may also reduce post-procedural hypotension, which has been shown to be one of the determinants of contrast-induced AKI [2]. In this study, we sought to investigate whether a systematic distal radial approach using 5 Fr guiding sheaths was a safe and effective alternative to the transfemoral approach in performing CAS.

## 2. Materials and Methods

From July 2020 to October 2022, 2 interventional operators at our center (GDG, LS) systematically performed CAS using a 5 Fr distal radial approach in consecutive patients with an established indication for CAS. Both operators had significant experience in transfemoral CAS (>50 patients/year) but only episodic experience in TRCAS. 

All patients underwent angio-CT of the supra-aortic vessels to confirm the indication. However, operators performing the procedure were blind to the aortic arch anatomy.

Exclusion criteria were a non-palpable distal radial artery; known anatomic contraindications (i.e., high take-off radial artery, previous painful catheterization from the radial access); and estimated glomerular filtration rate of <30 mL/min.

Patients were classified as symptomatic if they had experienced a recent transient ischemic attack, stroke, or transient monocular blindness ipsilateral to the study artery in the preceding 180 days before enrollment. Otherwise, they were classified as asymptomatic.

Informed consent as approved by the local Ethics Committee for the use of personal data was obtained from each patient.

### 2.1. Interventional Procedure

**Figure 1:**
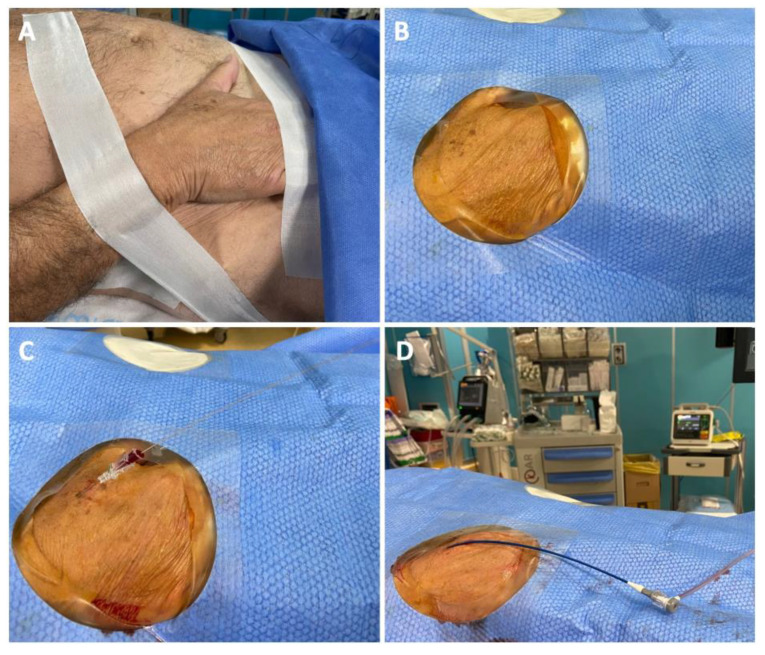
Distal radial access preparation. (**A**) The wrist of the patient is positioned in the neutral semi-prone position at the level of the omolateral groin and fixed with tape; (**B**) Disinfection and sterile draping; (**C**) Single wall puncture and 5 Fr sheath introduction (not shown); (**D**) The 5 Fr short sheath is exchanged with a 5 Fr × 90 cm guiding sheath.

Right or left vascular access was obtained without ultrasound guidance in all patients. The wrist of the patient was positioned in the neutral semi-prone position at the level of the omolateral groin and fixed with tape. After disinfection and sterile draping, local anesthesia was administered with 1–2 mL of Carbocaine at the level of the skin and periarterial soft tissue. Single wall puncture was preferable. Wiring was performed with the J-shaped wire of the 5 Fr introducer sheath or with a 0.014” hydrophilic wire. Fluoroscopy was performed to ascertain that the wire would advance in the forearm and not in the hand anastomotic arches. A small incision of the skin was performed adjacent to the wire before 5 Fr sheath insertion (RadifocusTM Introducer II, Terumo Corporation, Tokyo, Japan). Subsequently, a cocktail of 2.5–5 mg of Verapamil and 5000–6000 UI of UFH was carefully administered through the sheath. Figure 1 depicts the various steps for obtaining distal radial artery access for CAS.

A stepwise approach from least invasive to most invasive was ensured for vascular access. If distal radial access could not be obtained despite a palpable pulse before the procedure, the operators had to cross over to a proximal radial access with 5 Fr sheaths. If radial failure occurred due to severe spasm or inability to cannulate the common carotid artery due to excessive tortuosity, the operators had to cross over to a 5 Fr or 6 Fr femoral access, as preferred.

**Figure 2:**
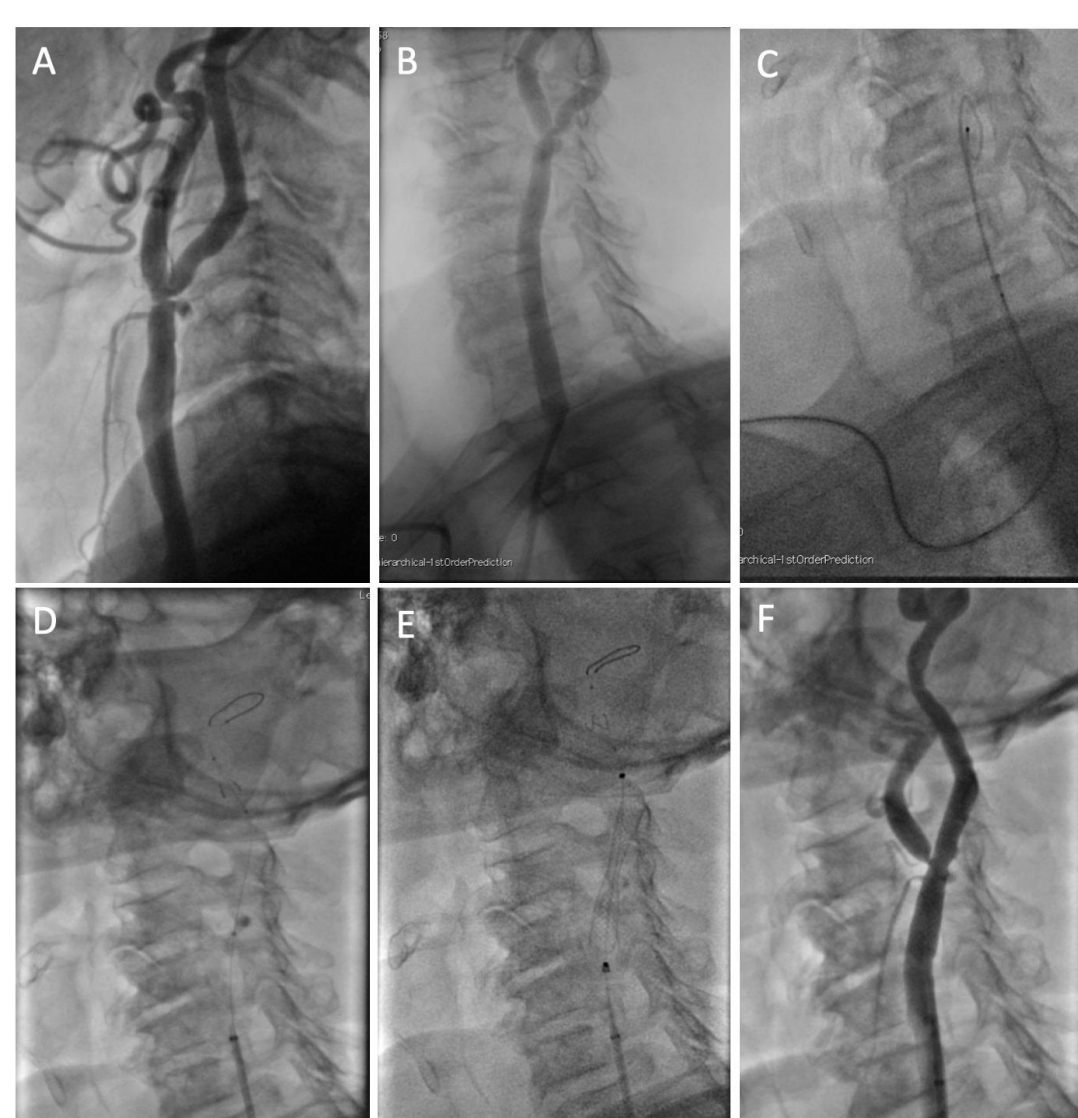
Example case of a patient with concomitant severe left internal and external carotid stenosis. (**A**) Baseline angiogram; (**B**) We performed a roadmap view from the diagnostic Simmons catheter. We failed to advance a hydrophilic wire into the ECA; (**C**) We shaped the tip of the stiff wire as a pigtail and positioned it in the distal part of the CCA, then advanced a 5 Fr × 90 cm guiding sheath in the CCA; (**D**) We crossed the stenosis of the ICA with a wire and positioned a distal embolic protection system in the distal part of the extracranial ICA; (**E**) We implanted a self-expanding stent and then postdilated it with a balloon; (**F**) Final angiogram.

Angiography of the supra-aortic vessels was performed with 5 Fr Judkins right or with Simmons-2 125 cm long diagnostic catheters. Subsequently, either the Anchor or the Telescoping technique was used to position the 5 Fr introducer sheath in the common carotid artery (CCA). The former, similarly to the technique used for femoral access, consists in advancing the diagnostic catheter in the external carotid artery (ECA) over a 0.035” hydrophilic wire that is then exchanged for a 0.035” stiff wire (Hi-Torque Supra Core, Abbott, Chicago, IL, USA). Subsequently, the diagnostic catheter and short sheath are removed, and a 5 Fr × 90 cm introducer sheath is advanced in the distal CCA over the stiff wire. The Telescoping technique, particularly useful for cannulating right CCAs with a steep angle (<45°) or left non-bovine CCAs, consists in advancing the introducer sheath over a 125 cm Simmons 2 catheter positioned in the ECA over a hydrophilic or a stiff wire for support (2). When a severe stenosis of the ECA was also present, the tip of the stiff wire was shaped as a pigtail to increase support and positioned in the distal CCA (Figure 2).

Once the 5 Fr × 90 cm introducer sheath (DestinationTM, Terumo, Japan; Halo ONETM, BD, USA; Flexor^®^, Cook Medical, Bloomington, IN, USA) was positioned in the distal CCA, the ICA stenosis was crossed with a 0.014” guidewire, and a distal embolic protection was placed as distally as possible in the extracranial ICA (Emboshield NAV 6TM, Abbot, Chicago, IL, USA; Spider FXTM, Medtronic, Minneapolis, MI, USA). 

Predilation with a balloon was performed only if deemed necessary by the operators. To allow good visualization of the stenosis in a 5 Fr environment, a fluoroscopic roadmap was performed before stent insertion. Either a closed-cell (Wallstent, Boston Scientific, Marlborough, MA, USA) or an open-cell (Mer, Balton, Warsaw, Poland) stent was implanted according to operators’ preference.

Postdilation was performed with a 5.0 or 5.5 × 20 mm balloon. The filter was retrieved, and final angiography was obtained in at least 2 projections.

Distal radial hemostasis was obtained with the Prelude SYNC DISTALTM radial compression device (Merit Medical, South Jordan, UT, USA) upon removal of the sheath. Patent hemostasis was achieved by slow release of air from the initially fully inflated balloon until a small amount of brisk blood flow was visualized from the puncture site. Then, the balloon was re-inflated with 2 mL of air.

All patients were treated with saline infusion at 100–150 mL/h for 24 h after the procedure. Patients with baseline serum creatinine > 1.2 mg/dL were hydrated with saline at a similar rate for 12 h before the procedure, according to institutional protocol.

All patients were pre-treated with Clopidogrel and Aspirin. Those who were naïve to antiplatelet therapy were loaded with 300 mg of Clopidogrel o.d. and/or 250 mg of Aspirin i.v. 12–24 h before the procedure. Oral anticoagulation with Apixaban, Edoxaban, Dabigatran, or Rivaroxaban was suspended 48 h before the procedure. After discharge, patients in need of anticoagulation received triple antithrombotic therapy for 1 week, then aspirin was suspended.

### 2.2. Endpoints

The main endpoints of the study were procedural success via distal radial and via proximal or distal radial access. The learning curve for the procedure was evaluated by comparing procedural success in the first half of patients versus the second half of patients enrolled.

Procedure length, contrast dose, X-ray time and dose were evaluated. Proximal radial artery patency was evaluated via Doppler ultrasound at 24 h and by the presence of a palpable radial pulse at 30 days.

AKI was defined as an increase in serum creatinine ≥ 150% or ≥0.3 mg/dL within 48 h [9].

Clinical outcomes were collected in-hospital and 30 days after the procedure through outpatient visits. Minor stroke was defined as an ischemic attack with symptoms lasting more than 24 h and a modified Rankin Scale (mRS) ≤ 2. Major stroke was defined as a mRS > 2 [10]. Myocardial infarction was defined as a hospital admission for non-ST-elevation myocardial infarction or ST-elevation myocardial infarction during the first 30 days after the procedure.

### 2.3. Statistical Analysis

All analyses were performed with SPSS 27.0 (IBM Inc., New York, NY, USA).

Continuous variables are reported as medians and interquartile ranges. Categorical variables are reported as frequencies and percentages. Comparisons between continuous variables were performed with the Student’s *t*-test. Comparisons between categorical variables were evaluated with the Pearson’s χ^2^ test.

Probability values were two-sided, and values of *p* < 0.05 were considered significant.

## 3. Results

### 3.1. Procedural and Clinical Outcomes

A total of 51 patients were enrolled in the prospective registry. Clinical characteristics are reported in Table 1. In 29 (57%) patients, coronary angiography was performed during the same procedure, as per clinical indication. In all of those cases, coronary angiography was performed prior to bilateral carotid angiography and stenting.

Procedural characteristics are reported in Table 2. CAS was effectively performed via distal radial access in 45 patients (88%). In two cases (4%), operators were unable to puncture the distal radial artery and successfully performed the procedure via the proximal radial artery with 5 Fr sheaths. Overall radial artery success was then 92%. In four cases (8%), a switch to femoral access with 5 Fr or 6 Fr sheaths was necessary for the following reasons: severe spasm of the brachial artery (*n* = 2); inability to cannulate the CCA with the sheath due to excessive tortuosity (*n* = 2). In three of these cases, the chosen initial access site was the left radial artery. All successful TRCAS were performed via the right radial artery.

Procedure time was 50 (42, 63) min for the overall procedure, whereas it was 47 (36, 55) min after removing time from effective puncture of the distal radial to the end of coronary angiography in patients in which it was performed.

X-ray time was 23 (17, 27) min; X-ray dose was 445 (202, 796) mGy; dose area product (DAP) was 44 (27, 75) Gy × cm^2^. After removing the contribution of coronary angiography, X-ray time was 18 (14, 24) min; X-ray dose was 398 (177, 612) mGy; DAP was 36 (22, 61) Gy × cm^2^.

Contrast dose administered was 120 (100, 140) mL for the overall procedure and 90 (70, 115) mL excluding coronary angiography.

Proximal radial artery was occluded in one patient 24 h after the procedure and at 30 days (2%). In this patient, the 5 Fr introducer had to be exchanged for a 7 Fr femoral introducer in order to accommodate a 5 Fr × 130 cm long catheter over a 0.018” guidewire. No access site hematoma was reported. AKI was observed in three (6%) patients 24 h–48 h after the procedure. No patient required renal replacement therapy.

Clinical outcomes in hospital and at 30 days are reported in Table 3. Hospital length of stay was 2 days in 44 patients (86%). Seven patients were hospitalized from 3 up to 10 days. In one case (2%), minor stroke was observed periprocedurally, following the rupture of the postdilation balloon. It presented with hyposthenia of the right arm and leg, with complete regression of the symptoms 3 days after the procedure. No other stroke was reported at 30 days. No death or MI were reported in hospital and at 30 days.

### 3.2. Learning Curve

The learning curve is reported in Table 4. Distal radial CAS was successfully performed in 20 patients out of the first 25 patients enrolled (80%), and in 25 of the last 26 patients enrolled (96%; *p* = 0.07). A switch to proximal radial access was necessary in two patients (8%) of the first 25 enrolled, whereas it was never necessary in the last 26 patients. A switch to femoral access was performed in three patients (12%) of the first 25 and in one patient of the last 26 (3.8%).

A significantly lower contrast dose was administered in the last 26 patients compared to the first 25 enrolled (110 (70, 140) mL vs. 120 (107, 150) mL, respectively; *p* = 0.045).

Numerically lower values of procedure time, X-ray time, X-ray dose, and DAP were observed in the second half of patients enrolled, although the difference was not statistically significant.

## 4. Discussion

In this single-center prospective registry, we sought to investigate whether a TRCAS performed with a systematic distal radial 5 Fr approach, irrespective of supra-aortic arch anatomy, was a safe and effective alternative to transfemoral CAS.

We found that:

- Success rate for distal radial CAS was around 90%, with a relatively short learning curve;
- The rate of RAO was very low, in line with data on distal radial access for coronary interventions;
- The procedure was safe, with low rates of clinical events at 30 days.

The rationale of this prospective registry was to try to reduce patients’ discomfort and RAO to a minimum in patients who are often subject to multiple catheterizations during their lifetime. In addition, the earlier mobilization of patients could reduce post-procedural hypotension and thus, the cerebral and renal complications that go along with it [11].

To accomplish this, a stepwise approach from the least to the most invasive procedure was ensured in our protocol.

In particular, 5 Fr radial access was preferred because it has shown a lower rate of RAO and vascular complication as compared with 6 Fr, and its size better suits the smaller size of the distal radial artery, potentially minimizing the incidence of spasm [12].

Alongside this, the interest in distal radial access, at first promoted by a few enthusiasts on social media [13], has flourished after various studies reported a lower rate of RAO compared to conventional radial access [3], whose post-procedural occlusion rate is around 5–7% [14]. Some skepticism arose after the randomized DISCO-RADIAL trial did not show significant difference in RAO rates between distal and proximal radial access for coronary intervention [4]. However, a very strict patent hemostasis protocol was implemented in the proximal radial group [15], and researchers were able to obtain very low RAO rates in both proximal and distal radial artery groups (respectively, 0.9% and 0.3%; *p* = 0.29). It is debatable whether such a strict hemostasis protocol could be easily implemented in real life. However, a recent meta-analysis of 14 trials [5] showed that despite a longer puncture time, there were significantly lower rates of RAO and major forearm hematomas with distal as compared to conventional radial access.

In our experience, TRCAS via the distal radial approach was successfully performed in 88% of the patients, while overall transradial success rate was 92%, similar to previous literature on radial access for CAS. In the RADCAR trial [16], the only randomized trial to date comparing the radial and femoral approach for CAS, crossover rate was 10% in the radial arm and 1.5% in the femoral arm. In a subsequent meta-analysis of seven studies including 723 patients [17], success rate for TRCAS was also 90.8%. Interestingly, in our registry, success rate for distal TRCAS rose from 80% to 96% in the second half of patients enrolled, indicating a clear learning curve for the procedure. Moreover, while no significant difference was found in terms of procedure time and X-ray exposure, significantly less contrast medium was administered in the second half of patients enrolled. Notably, to be in line with real life practice, in our registry, no ultrasound guidance was used to puncture the distal radial artery, indicating no real need for ultrasound after a learning curve period.

In our study, RAO occurred only in one patient (2%), similarly to recent data on distal radial access for coronary interventions [3].

Despite being a more technically challenging procedure, radiation exposure and DAP were in line with previous reports on transfemoral CAS [18].

AKI has been reported much more frequently in CAS procedures as compared to coronary interventions. A study by Donahue et al. [19] reported an incidence of AKI of 21% in patients with pre-existing kidney disease undergoing CAS. Radial access has already been shown to reduce renal injury in coronary patients. In fact, a pre-specified subanalysis of the randomized MATRIX trial [7] showed a significant reduction in AKI in patients who received coronary interventions from radial as compared to femoral access. These results were later confirmed by meta-analyses of non-randomized and randomized trials [8]. The mechanisms of such renal injury reduction are a reduction in bleedings, vascular complications and renal cholesterol embolization. In addition to coronary procedures, CAS is afflicted with a high rate of peri-procedural and post-procedural hypotension that has been recognized as an important predictor of AKI [2,11]. Since early mobilization has been shown to significantly increase short-term heart rate and blood pressure, radial artery access for CAS has the potential to reduce all the mechanisms involved with AKI at once [20].

In our registry, only 6% of patients experienced AKI, similarly to coronary procedures. Studies with a larger sample size are necessary to further investigate this issue.

Clinical outcomes were favorable in-hospital and at 30-days. Only one minor stroke was observed, whereas no death or MI were reported.

Although the small sample size limits the comparability of the results, these outcomes are in line with current literature for the femoral approach. A prospective registry of eight high-volume European centers, including our own, showed a 30 day incidence of stroke of 1.12% and of death of 0.25% [21]

This study has various limitations. This is a prospective single-center, single-arm observational registry, with the intrinsic shortcomings of potentially limited external validity and intrinsic bias. The sample size was relatively small. Nevertheless, this study represents the first prospective report on the use of distal radial access for carotid artery stenting. The occurrence of hypotension and bradycardia during the procedure was not systematically collected. Not all stents and measures are compatible with 5 Fr guiding sheaths.

## 5. Conclusions

In our experience, distal TRCAS using 5 Fr catheters was a safe procedure with a high success rate. Distal radial artery access for CAS had a relatively short learning curve in operators familiar with CAS, irrespective of aortic arch anatomy. TRCAS has the potential to become the default access for CAS to reduce vascular complications and patients’ discomfort. Snuffbox access could represent a viable alternative, allowing for reduction of RAO and hemostasis time, in order to save radial access for future procedures and potentially perform CAS without discontinuation of anticoagulation.

## Figures and Tables

**Table 1:** Clinical characteristics.

	Overall (*n* = 51)
Age, years	72 (67, 77)
Male sex, *n* (%)	33 (65%)
Current smoker, *n* (%)	13 (25%)
Former smoker, *n* (%)	12 (23%)
Dyslipidemia, *n* (%)	34 (67%)
Hypertension, *n* (%)	40 (78%)
Diabetes mellitus, *n* (%)	18 (35%)
Insulin therapy, *n* (%)	6 (12%)
Lower limb PAD, *n* (%)	6 (12%)
Coronary artery disease, *n* (%)	28 (55%)
Oral anticoagulation, *n* (%)	5 (10%)
Previous MI, *n* (%)	8 (16%)
Previous PCI, *n* (%)	15 (30%)
Previous CABG, *n* (%)	5 (10%)
Previous stroke, *n* (%)	8 (16%)
Symptoms, *n* (%)	11 (22%)
Serum creatinine, mg/dL	1.0 (0.8, 1.3)

PAD, peripheral artery disease; MI, myocardial infarction; PCI, percutaneous coronary intervention; CABG, coronary artery bypass grafting.

**Table 2:** Procedural characteristics and outcomes.

	Overall (*n* = 51)
Distal radial access site, *n* (%)	Right: 48 (94%) Left: 3 (6%)
CAG during the same procedure, *n* (%)	29 (57%)
Aortic arch type, *n* (%) - Type 1 - Type 2 - Type 3 - Bovine arch - Not available	21 (42%) 8 (16%) 7 (14%) 11 (22%) 3 (6%)
Right ICA culprit stenosis, *n* (%)	34 (67%)
Diameter stenosis (%)	80 (75, 85)
Contralateral stenosis > 50%, *n* (%)	8 (16%)
Sheath size, *n* (%)	5 Fr: 50 (98%) 6 Fr: 1 (2%)
Diagnostic catheter, *n* (%)	JR 4: 8 (16%) JL 3.5: 3 (6%) Sim 1: 8 (16%) Sim 2: 32 (63%)
Guiding sheath, *n* (%)	Destination 5 Fr × 90 cm: 27 (53%) Destination 6 Fr × 90 cm: 1 (2%) Halo One 5 Fr × 90 cm: 20 (39%) Flexor 5 Fr × 70 cm: 2 (4%) Micro 5 Fr × 130 cm: 1 (2%)
Distal embolic protection	Emboshield NAV6: 46 (90%) Spider FX 6 mm: 2 (4%) Spider FX 7 mm: 3 (6%)
Predilatation, *n* (%)	8 (16%)
Stent implanted, *n* (%)	Wallstent: 24 (47%) Mer: 27 (51%) * X-Act: 1 (2%)
Postdilation balloon, *n* (%)	5.0 × 20 mm: 27 (53%) 5.5 × 20 mm: 24 (47%)
Procedure time, min	50 (42, 63)
Procedure time, excluding CAG, min	47 (36, 55)
X-ray time, min	23 (17, 27)
X-ray time, excluding CAG, min	18 (14, 24)
X-ray dose, mGy	445 (202, 796)
X-ray dose, excluding CAG, mGy	398 (177, 612)
DAP, Gy × cm^2^	44 (27, 75)
DAP, excluding CAG, Gy × cm^2^	36 (22, 61)
Contrast dose, mL	120 (100, 140)
Contrast dose, excluding CAG, mL	90 (70, 115)

* In one patient 2 Mer stents were implanted. CAG, coronary angiography; ICA, internal carotid artery; DAP, dose area product.

**Table 3:** Clinical outcomes.

Procedural Outcomes	
Procedural success, *n* (%)	51 (100%)
Crossover to proximal radial, *n* (%)	2 (4%)
Crossover to femoral, *n* (%)	4 (8%)
Access site hematoma	0 (0%)
RAO at 24 h	1 (2%)
RAO at 30 days	1 (2%)
AKI at 24–48 h	3 (6%)
Length of hospitalization, days	2 (2, 2)
**In-hospital outcomes**	
Death, *n* (%)	0 (0%)
Myocardial infarction, *n* (%)	0 (0%)
Minor stroke, *n* (%)	1 (2%)
Major stroke, *n* (%)	0 (0%)
**30-day outcomes**	
Death, *n* (%)	0 (0%)
Myocardial infarction, *n* (%)	0 (0%)
Minor stroke, *n* (%)	1 (2%)
Major stroke, *n* (%)	0 (0%)

RAO; radial artery occlusion; AKI, acute kidney injury.

**Table 4:** Learning curve.

	First Half (*n* = 25)	Second Half (*n* = 26)	*p* Value
Radial success			
Distal radial success	20 (80%)	25 (96%)	0.07
Procedure time, min	57.8 (43.5, 63.5)	49.0 (39, 61.5)	0.47
X-ray time, min	24.6 (18.6, 27.4)	19.8 (16.1, 27.78)	0.49
X-ray dose, mGy	452 (152.5, 800.5)	438 (209, 776)	0.26
DAP, Gy × cm^2^	46.1 (30.2, 78.9)	43.4 (25.3, 70.6)	0.56
Contrast dose, mL	120 (107, 150)	110 (70, 140)	0.045

DAP, dose area product.

## Data Availability

Anonymized data are available, following motivated request to the corresponding author.
